# Neuromodulation influences synchronization and intrinsic read-out

**DOI:** 10.12688/f1000research.15804.2

**Published:** 2018-12-02

**Authors:** Gabriele Scheler

**Affiliations:** 1 Carl Correns Foundation for Mathematical Biology, Mountain View, CA, 94040, USA

**Keywords:** neuromodulation, synchronization, network topology, synaptic efficacy, intrinsic excitability, asynchronous, activation function

## Abstract

**Background**: The roles of neuromodulation in a neural network, such as in a cortical microcolumn, are still incompletely understood. Neuromodulation influences neural processing by presynaptic and postsynaptic regulation of synaptic efficacy. Neuromodulation also affects ion channels and intrinsic excitability.

**Methods**: Synaptic efficacy modulation is an effective way to rapidly alter network density and topology. We alter network topology and density to measure the effect on spike synchronization. We also operate with differently parameterized neuron models which alter the neuron's intrinsic excitability, i.e., activation function.

**Results**: We find that (a) fast synaptic efficacy modulation influences the amount of correlated spiking in a network. Also, (b) synchronization in a network influences the read-out of intrinsic properties. Highly synchronous input drives neurons, such that differences in intrinsic properties disappear, while asynchronous input lets intrinsic properties determine output behavior. Thus, altering network topology can alter the balance between intrinsically vs. synaptically driven network activity.

**Conclusion**: We conclude that neuromodulation may allow a network to shift between a more synchronized transmission mode and a more asynchronous intrinsic read-out mode. This has significant implications for our understanding of the flexibility of cortical computations.

## Introduction

In this paper we present a realistic network model, akin to a cortical microcolumn ^1– 4^, and investigate its properties under the assumption of fast synaptic and intrinsic modulation as evidenced by neuromodulation ^5^. We hypothesize that rapid synaptic efficacy changes allow a network to operate with different topologies, and that network topology is a decisive factor towards creating and sustaining synchronized inputs vs. producing asynchronous input.

We have previously shown for a conductance-based neural model of striatal medium spiny neurons that neuronal heterogeneity expressed by the contribution of individual ion channels (such as delayed rectifier potassium channels or GIRK channels) may still result in uniform responses, if the neurons are driven with highly correlated synaptic input. If the same neurons are driven by more asynchronous, distributed synaptic input, the heterogeneity is manifest in the response patterns, i.e. the spike rates and the timing of the spikes (see 6). These results were achieved using conductance-based point neurons ^6^. Here we use two-dimensional neural models ^7^ to further investigate the effect and determine its significance in the context of a cortical neural network.

Due to Hebbian learning ^8, 9^, under normal conditions synaptic weights follow a lognormal distribution, which results in graphs with a heavy tail degree distribution. Degree modification by rapid synaptic efficacy changes would not only allow for alterations to the density, but also the topology of the connecting graph. In this paper we examine the hypothesis that such changes in network topology actually occur, driven by neuromodulatory effects on presynaptic release or postsynaptic response ^5, 10– 12^. We analyze this situation with two example graphs, and we also perform further analysis to show that there is a continuum of graphs which can be reached by rapid synaptic changes.

## Methods

### Conductance-based neuron model and synaptic input

The conductance-based neural model of a striatal medium spiny neuron is described in detail in 6. The membrane voltage *V _m_* is modeled using the equation

$${\dot{V}}_{m}=-\frac{1}{C}\left[\mu_{1}(I_{\text{1}})+\mu_{2}(I_{\text{2}})+...+\mu_{n}(I_{n})-I_{syn}\right],\;(1)$$

where the *I _i_* are the currents, induced by the individual ion channels. Variability of the neuron is modeled by modifications to *µ _i_*. This model includes ion channels for Na (INa), K (IK), slow A-type K channels (IAs), fast A-type K channels (IAf), inward rectifying K channels (IKir), L-type calcium channels (ICaL), and the leak current (I _leak_). The definition of all parameters and the dynamics of the ion channels can be found in 6.

For the experiments in this paper, we use only a single channel as an example for the variability that can be induced by neuromodulatory changes. We chose the slow A-type K channels as in 6. The total current contribution for this channel is *µ*_IAs_ where *µ* was selected between 1.0 and 1.5, a variability by *±*25%.

In order to illustrate the variability in neuron behavior, we excited the neuron model by input signals, resembling two kinds of synaptic input: uncorrelated and correlated. These signals were generated by superposition of excitatory and inhibitory spikes from individual Poisson-distributed spike trains (50 excitatory and 10 inhibitory), and biased Gaussian background noise. The details can be found in 6. The amount of pairwise correlation in these spike trains governs the type of input signal. A high correlation factor was used in order to generate sequences which have short periods (10–15ms) of high activity.

### Heterogeneity in a two-dimensional model 

In order to do large-scale simulations we needed to employ a simple, computationally tractable neuron model. We used a two-dimensional model of a neural oscillator (cf. 7), and employed an instantiation of the model with parameters fitted to the general properties of cortical pyramidal neurons ^13^ as a generic model (g). The model consists of an equation for the membrane model *v* ( Equation 2), fitted to experimental values for cortical pyramidal neurons, and an equation for a gating variable *u* ( Equation 3).

$$\dot{v}=0.04v^{\text{2}}+5v+\text{140}-u-I_{syn}\;(2)$$

$$\;\begin{array}{l} \dot{u}=a(bv-u)\;(3)\\ b=0.2\\ a=0.02 \end{array}$$

When the neuron fires a spike (defined as *v*( *t*) = 30 *mV*), *v* is set back to a low membrane potential *v* := *c*; *c* = *−*65.8 *mV* and the gating variable *u* is increased by a fixed amount *d* ( *u* := *u* + *d*; *d* = 8) (cf. 13). This formulation allows for a very simple neuron model, which avoids the explicit modeling of the downslope of the action potential, and rather resets the voltage. Time-dependence after a spike is modeled by the gating variable *u*.

Neuronal heterogeneity is achieved by systematic variation of inactivation parameters. By varying *d*, we can vary the inactivation dynamics of the model after a spike, by varying *a* we vary the activation/inactivation dynamics for *u*. In this way, we can model neuronal variability of activation/inactivation dynamics, which is sufficient to model frequency-selectivity as a stored intrinsic property. The parameters used in this paper for different neuron types are listed in Table 1.

**Table 1.** Parameters for different neuron types(cf. 13).

name	*a*	*b*	*c*	*d*
generic	0.02	0.2	-65	8
type1	0.025	0.2	-65	6
type2	0.02	0.2	-65	9
type3	0.015	0.2	-65	12
type4	0.015	0.15	-65	14
type5	0.022	0.3	-65	14
type6	0.022	0.3	-65	9.5

### Graph properties 

We created graphs of *N* (= 210) excitatory neurons, and *K* ( *≈* 1900) excitatory connections. For the excitatory neurons, we use randomly connected graphs (N,K) with different width σ* = *e ^σ^* (σ = standard deviation) of the degree distribution. This corresponds to normal (Gaussian) to lognormal graphs with different widths and length of the heavy tail. We model inhibition by Poisson-distributed inhibitory synaptic input directly onto excitatory neurons.

We use specific instantiations of these graphs (RG, LG1) for the simulations. Table 2 shows global graph characteristics for the Gaussian graph (RG), the lognormal graphs (LG1), and intermediate graphs LG2, LG3, and LG4.

**Table 2.** Graph Properties for Model Networks (Excitatory Connections).

Property	RG	LG1	LG2	LG3	LG4
N no of nodes	210	210	210	210	210
K connections	1880	1924	1895	1920	2050
d density	0.042	0.043	0.043	0.043	0.047
indegree	8.95[2...17]	9.5[0...26]	9[0...24]	9.14[0...30]	9.75 [0...25]
outdegree	8.95[4...18]	9.5[0...56]	9[0...58]	9.14[0...53]	9.75 [0...54]
cluster index	0.04	0.068	0.064	0.062	0.08
mean path length	2.66	2.41	3.7	3.72	2.32
width σ*	1.44	2.89	2.5	2.71	2.71
synchronization *s*	0.11	0.32	0.26	0.31	0.34

The rewiring algorithm used to change the properties of a graph G is a greedy algorithm, which iteratively selects the node with the highest degree. One of its edges is then rewired to random nodes with lower degrees, decreasing σ*. The algorithm terminates, when the value of σ* falls below a given threshold.

### Definitions

We define synchronization *s* in a network by pairwise correlations: for each neuron *n _i_*, we count, for each other neuron *n _j_*, the number of spikes which occur within a window *W* ( *W* = 10 *ms*) of *n _i_*’s spike events, divided by the total number of spikes for *n _i_*. More precisely, for each neuron, we bin all firing events into 5ms bins. We then count the number of spikes emitted by other neurons, which fire in a 10ms window around the (start) of the bin. The synchronization *s* is then the average over all neuron pairs in the network:

$$s=\frac{1}{N\left(N-1\right)}\sum_{j}\frac{1}{S_{j}}\sum_{i}B_{ij},$$

where *B _ij_* is the number of spikes that neurons *i* and *j* have in common within a moving window of *W* = 10 *ms* during the entire measuring time. *S _j_* is the number of spikes of neuron *j* during the entire measuring time.

### Simulation tools

All simulations were performed with the software tool CNeuroSim, which is implemented in Matlab (R2016b) and C, and available at https://doi.org/10.5281/zenodo.1164096.

## Results

### Conditional expression of intrinsic excitability in conductance-based models

We show how we can model gain as a stored intrinsic property, defined as the average spike rate in response to constant input (constant input in A / average spike rate in Hz). We used a full ion channel based model (the MSN model ^6^), with variation in the slow A-type potassium channel (IAs). This ion channel was used as an example for the conductance-based model ^6^. Neuromodulation often affects just one type of ion channel, and a total variations of 30–50% in ion channel efficacy have been typically found.

**Figure 1.**
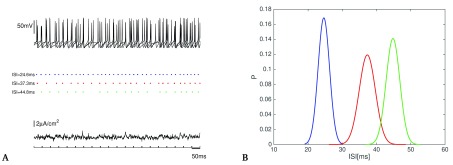
**A**. Frequency response of 3 conductance-based MSN model neurons with variable scaling of IAs to uncorrelated input **B**. Probability distributions of ISIs. We see a clear separation of frequency responses.

In Figure 1A, we show the response of individual, unconnected MSN model neurons with a scaling of *µ*_IAs_ = 1.0, 1.3, 1.5 to a noisy input signal, derived from simulations of neural activity as uncorrelated Poisson-distributed spiking. The top panel shows the development of the membrane potential, *V _m_*, over time for all neurons. The middle panel shows the spike-train for each neuron with the mean interspike interval (ISI). The bottom panel shows the simulated synaptic input. The dots correspond to the spiking events for a single neuron 3 ( *µ*_IAs_ = 1.5). The resulting mean ISIs are 25, 37, and 45 ms. With a standard deviation of 6, 11, and 8, they are clearly distinguishable. This is also shown by the Gaussian distribution for the mean ISIs for each neuron type ( Figure 1B).

This model shows frequency-specificity as read-out of the relative contribution of the slow A-type potassium channel, indicated by the scaling factor *µ*_IAs_. The relative contribution of an ion channel corresponds to its density or distribution on the somato-dendritic membrane, or in some cases its specific localization at dendritic branch points. Experimental evidence has shown that this is a plastic feature for neurons.

**Figure 2.**
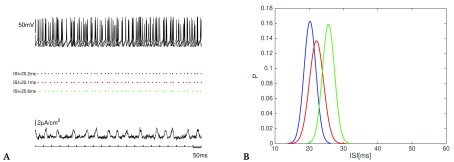
**A**. Frequency response of the same MSN model neurons as in Figure 1 to correlated input. **B**. Probability distribution of ISIs. We see overlapping of frequency responses.

We then employ highly correlated synaptic input, defined as in 6 (see *Methods*). We stimulate the same neurons with the correlated input and observe the spike pattern ( Figure 2). We can show that the frequency-specificity of the neuron disappears. Instead we see a time-locked spike pattern which is expressed by a similar spike frequency ( Figure 2A) and an overlap of the mean ISIs ( Figure 2B). What this experiment showed is that a stored intrinsic property, the gain, is available to the processing network in a conditional manner. The property is continually expressed, the differences in ion channel density persist. Depending on the mode of stimulation, however, this property is manifested as intrinsic gain, or it is obscured when a neuron is driven by strongly correlated input.

### Results for simplified model neurons

**Figure 3.**
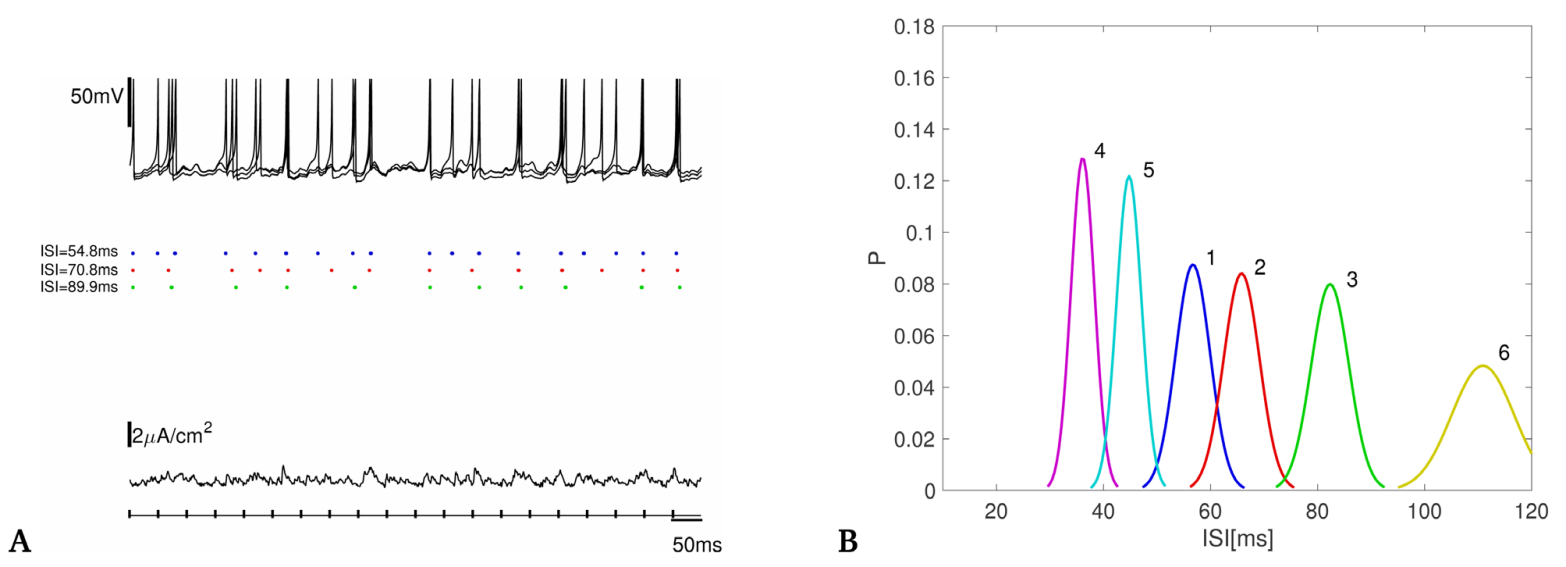
**A**. Spike response of the two-dimensional dynamic model neurons (1,2,3) to asynchronous input. **B**. Gaussian distributions of ISIs for six model neurons (1,2,3,4,5,6) to asynchronous input as in **A**. We see a clear separation of frequency responses for model neurons.

**Figure 4.**
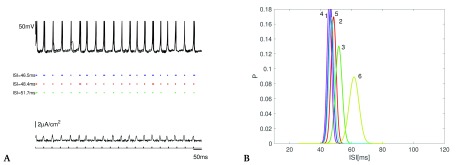
**A**. Spike response of the two-dimensional dynamic model neurons (1,2,3) to regularly timed, correlated input. **B**. Distributions of ISIs for six model neurons (1,2,3,4,5,6) to the same input as in **A**. We see strong overlapping of frequency responses, at about 50ms ISI, in accordance with the input. We notice that neuron 6 fires at lower frequencies than the input, it probably has a longer reset period, as seen in Figure 3B.

**Figure 5.**
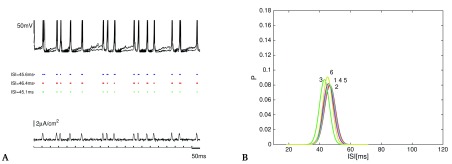
**A**. Spike response of the two-dimensional dynamic model neurons (1,2,3) to irregularly timed, correlated input. **B**. Gaussian distributions of ISIs for six model neurons (1,2,3,4,5,6). We see strong overlap of frequency responses.

To continue with exploring this property of model neurons, we switched to a simplified model neuron ^7^ and created a set of variations for this model (see *Methods*). We show the response of two-dimensional model neurons to asynchronous input in Figure 3, and to regular, synchronous input in Figure 4. In the first case, we have clearly separated frequencies, and in the second case, the ISIs are nearly identical with a narrow distribution. When we stimulate the neurons with irregular, but synchronous input, the ISIs become identical, but with a wider distribution to reflect the different duration of pauses between the synchronous stimulation ( Figure 5).

### Multiplexing synchronous and asynchronous input

**Figure 6.**
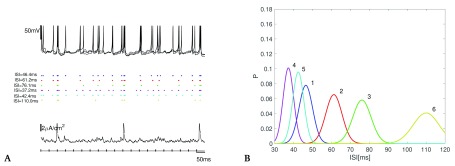
**A**. Multiplexed input and response of different model neurons (1-6). Three synchronous events are clearly represented in the spike pattern of all neurons. **B**. Mean ISIs for each neuron type. The separation of frequencies is kept. Compared to Figure 3B the standard deviation is somewhat higher because of the additional spikes caused by strong synchronous input.

We may also consider the question of whether a neuron can simultaneously respond to an input and read out its stored spike frequency. If there are single synchronous events, which interrupt ongoing spiking, can we recover the intrinsic properties for each neuron? In Figure 6 it is shown that this is possible. Figure 6A shows the input and the synchronous responses, and in Figure 6B we still see a clear separation of frequencies.

We conclude that we can multiplex asynchronous and synchronous input. It is also apparent that there needs to be a lower limit on the intervals between synchronous events that can be processed without disrupting intrinsic properties. This interval needs to be defined as functionally dependent on the intrinsic frequencies. In this case, it is 3/s for the synchronous events, with 10Hz for the slowest neuron.

### Synchronization depends on network topology

The simplified model neurons allow the creation of large networks of heterogeneous neurons and exploration of different topologies (cf. also 14, 15). We hypothesized that a lognormal graph, because of its hierarchical topology and the existence of hub neurons would lead to synchronization of action potentials – even with heterogeneous neurons – while a Gaussian topology would support asynchronous spiking behavior ^16, 17^. We define synchronization *s* in a network by pairwise correlation ( *Methods*). The spike frequency for each neuron type is assessed by the mean and standard deviation for ISIs, as before.

**Figure 7.**
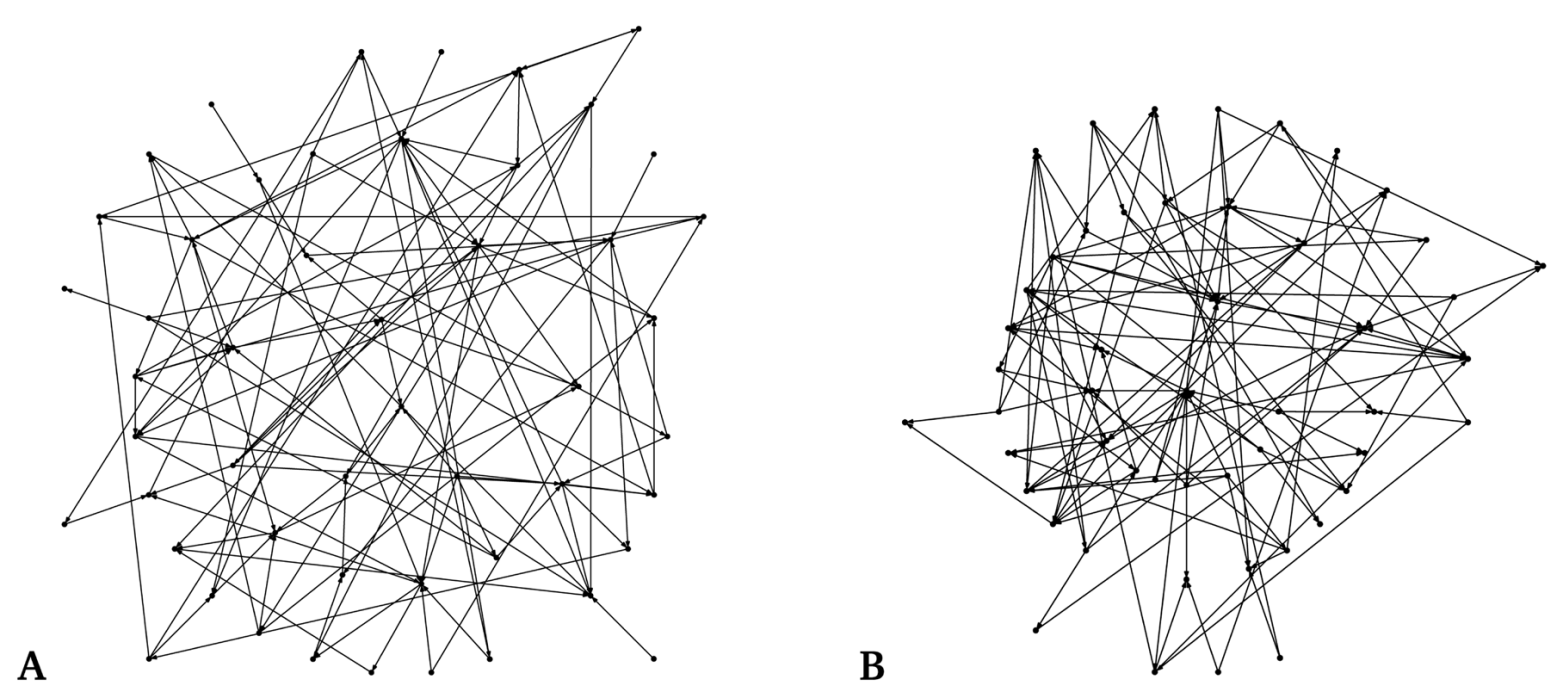
**A**. Part of a Gaussian Graph (RG), here for 50 neurons **B**. Part of a Lognormal Graph (LG1), for 50 neurons. The more regular, lattice-like structure of the Gaussian graph and the higher clustering and the appearance of highly connected ’hub’ neurons in the lognormal graph is apparent.

**Figure 8.**
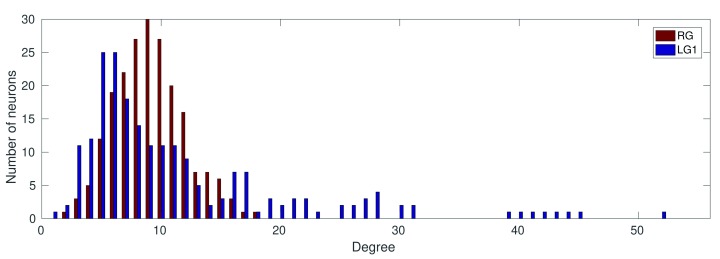
Degree histogram for the Gaussian Graph RG (red) and the lognormal Graph LG1 (blue). The LG has more neurons with few connections. It also has a heavy tail of neurons with 20 and more connections (’hubs’), which are lacking in the Gaussian graph.

We first use a randomly (Gaussian) connected graph (RG) with 210 neurons ( *N* = 210) and 1800 excitatory connections ( *K* = 1800). We employ 7 different neuronal types (1-6, plus the generic neuron *g*) with 30 Neurons each ( *Methods*). Figure 7A shows an excerpt of the graph structure. We can see that the graph is connected such that all neurons have a comparable number of connections. This is also apparent in Figure 8, where we can see a (narrow) normal distribution for connectivity for the Gaussian graph RG. Table 2 contains the usual graph characteristics.

*N* = 210 is about the size of a minicolumn or ensemble unit within a larger network with presumably dense interconnections ^18^. The maximal density *d* = *K/*( *N ×* ( *N −* 1)) in a cortical microcircuit is estimated at 0.1 for 10 ^4^ neurons, and 10 ^7^ synaptic connections, ^19^. With *≈* 50% of synapses internal to the network, *d* = 0.04 *−* 0.07 is a realistic value for internal connectivity ^18^. There is also a small background inhibition to all neurons present, implemented by 10% inhibitory neurons with Poisson-distributed firing and complete connectivity to excitatory neurons.

**Figure 9.**
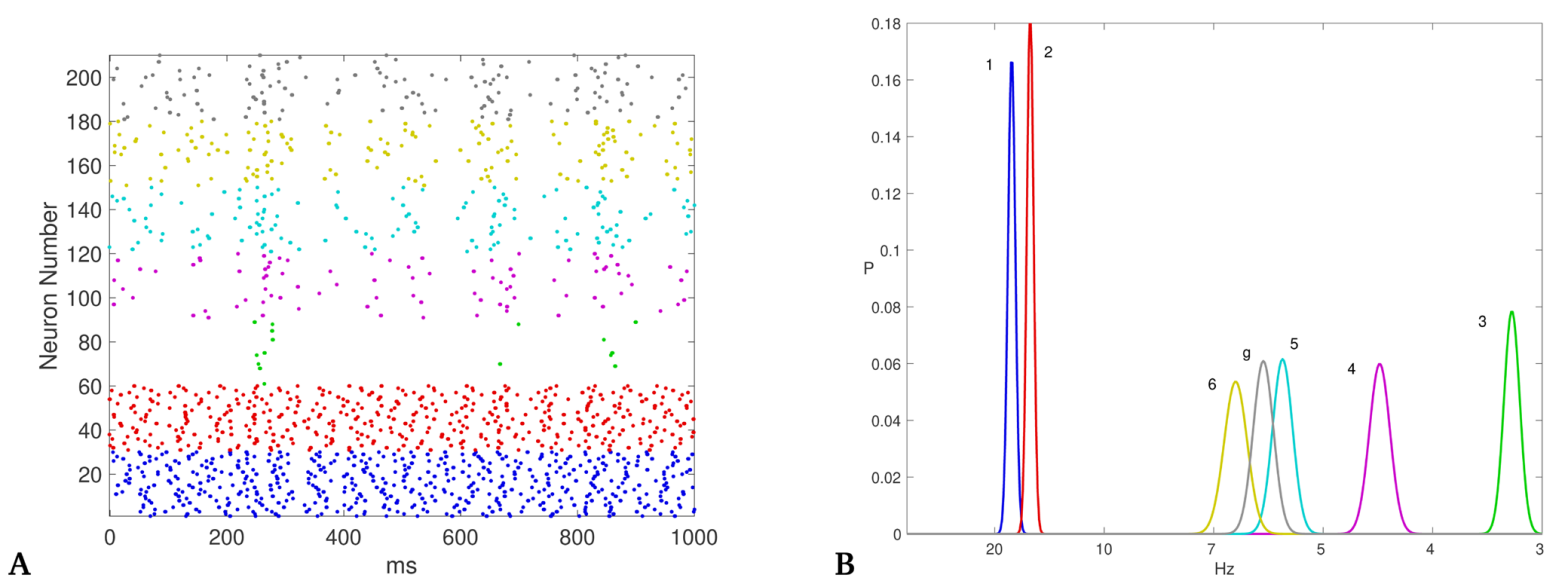
**A**. Asynchronous behavior in the Gaussian graph (RG) with variable neuron types. Groups of neuronal types with 30 neurons each are apparent in the rasterplot. Some structure is probably due to background inhibition. **B**. Average spikes/s with probability distributions for all neuronal types, with clear separation by frequency. Pairwise correlation is *s* = 0.11.

We now stimulate the graph by an initial stimulation to 10 randomly selected excitatory neurons (for about 1 second). In Figure 9A, we see highly asynchronous neuronal activity after 1s of stimulation. The pairwise correlation value *s* is low ( *s* = 0.11). Figure 9B shows that each neuronal type retains its own frequency, i.e., has its own typical ISI, separated from other neuronal types. We also notice that some neurons fire with low frequencies (5Hz) and others with higher frequencies (20Hz). Very low firing neurons (2Hz) which are typical for cortex are not represented in this model.

Next we changed the topology of the network to a graph with a lognormal distribution of connections (LG1), as shown in Figure 8. It used the same neurons ( *N* = 210) and approximately the same number of excitatory connections *K* = 1924 as before.

Figure 7B shows an excerpt of the lognormal graph structure from LG1. The connectivity structure seems much denser, because of ’hub’ neurons in the center of the graph. In Figure 8, we can see the wider distribution of degrees for the lognormal graph (blue), containing a number of nodes with high connectivity (’heavy-tailed distribution’). Presumably, those nodes are capable of synchronizing the network, because they can reach many neurons simultaneously. What is the effect on the presence of neural heterogeneity?

**Figure 10.**
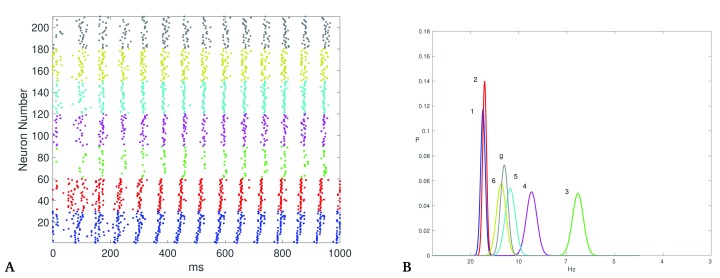
**A**. Synchronization in a lognormal graph (LG1) with variable neuron types. The rasterplot shows that different neuronal types respond uniformly. **B**. Frequency distributions. High overlap between neuronal types is apparent. Pairwise correlation in the graph is high with *s* = 0.32.

Figure 10 shows that a high amount of synchronization can be achieved in spite of heterogeneity of intrinsic frequency of model neurons. The rasterplot ( Figure 10A) shows the activity in LG1 with the same neurons and the same stimulation as before. The overall correlation, defined by pairwise correlation of neurons, is much higher (s=0.32). The distribution of ISIs in this case is strongly overlapping ( Figure 10B), similar to Figure 4B, where neurons were explicitly driven by highly synchronous input. This means that synchronization is dependent on the network topology, and a lognormal graph exhibits a higher tendency for pairwise synchronization. Also, that neuronal heterogeneity is apparent in an asynchronous network mode but is repressed in a synchronous firing mode.

### Dependence of synchronization on graph properties 

We could show that differences in intrinsic properties appear or become more prominent when there is less synchronicity in a network. In our model, the pairwise synchronicity *s* is dominated by the network topology, more precisely by the width of the degree distribution ranging from Gaussian to lognormal.

**Figure 11.**
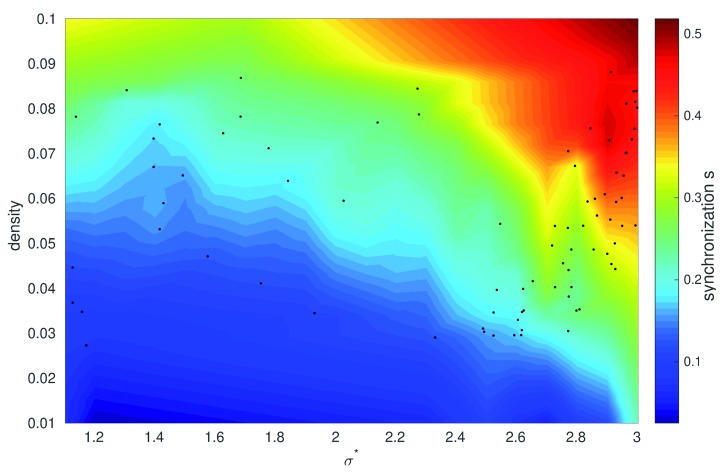
Synchronization *s* dependent on network topology: density and distribution width σ*. The experimentally attested distribution width for weights in cortical tissue ^8^ is σ* = 2 − 3.5, with a mean at 3. We achieve higher synchronization *s* in the lognormal region, also dependent on density, but no synchronization in the Gaussian (region of low width) ( σ* < 1.5), except close to maximal connectivity. Black dots signify actual measurements. There seem to be no abrupt transitions.

To confirm this observation we used a number of intermediate graphs and mapped the pairwise synchronization dependent on the degree distribution width σ* ( Figure 11). The graphs RG and LG1 that we used have values of σ* = 1.44 and σ* = 2.89 ( *Methods*). They have the same density, i.e., the same number of connections and neurons $\left(\tilde{0}.05\right)$. Additionally, we analyzed the dependence of synchronicity on the density of the graph between 0.01 and 0.1 ( Figure 11).

There is higher synchronization in the lognormal region, especially with σ ^*^ > 2.5, but no synchronization for Gaussian graphs. For heavy-tail graphs, synchronization depends linearly on the density between *d* = 0.03 *−* 0.08 ( *s* = 0.2 *−* 0.5).

**Figure 12.**
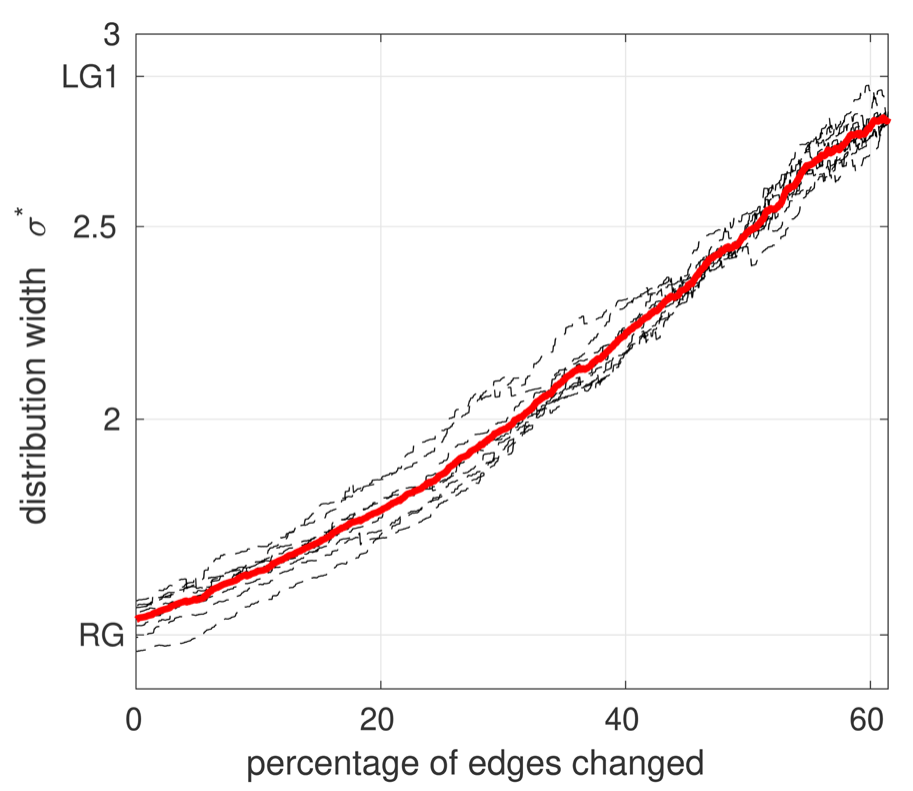
Transition between a lognormal graph and a Gaussian graph: For K=1900, N=210, d=0.43, mean over 10 trials, the percentage of edges changed to achieve a narrow degree distribution. The algorithm is not optimized (Methods) and overestimates the number of edges that have to be changed.

How are the different graphs related? We hypothesized that fast synaptic switching ^20^ by neuromodulation could change the network topology sufficiently to switch from a synchronous to an asynchronous regime. In Figure 12, we plot the number of edges that were changed to achieve different distribution width σ* of a graph. The algorithm used was a simple greedy algorithm ( *Methods*), which is suboptimal, i.e., overestimates the number of edges required. It appears that 30–50% of edges changed would be sufficient.

## Discussion

### Network Topology, Synchronization and Intrinsic Read-out

We employ a parameterizable two-dimensional neural oscillator model to encode different intrinsic excitability manifested by different frequency responses to constant input. What the experiments show is that a stored intrinsic property, the gain, is available to the processing network in a conditional manner: the gain is continually present, the differences in ion channel density persist. Depending on the mode of stimulation, however, this property is manifested as spike rate, or it is obscured when a neuron is driven by strongly correlated input. This is interesting because it shows a property of memory that synaptic plasticity lacks: the memory is not always ‘read-out’ in any processing step. It is conditional, it can be accessed or ignored depending on the state of the network. This seems to be an essential property of memory in any intelligent system.

Different statistical properties of synaptic input can be modeled by a variability in the correlation properties of input neurons. In a network model, this means that the overall correlation in the network determines what input a neuron receives. With a Gaussian degree distribution topology, correlation is low and neurons fire irregularly with their own preferred frequency. With a heavy-tailed, lognormal degree distribution topology, correlation is higher, and neurons fire when they receive correlated input, irrespective of intrinsic properties. I.e., driving neurons by correlated vs. uncorrelated input leads to uniform spiking behavior vs. read-out of stored differences in ion channel conductances.

### Inhibition

A restriction of the present model with respect to a biological simulation model is the simplified treatment of inhibition. However, experimental work shows that cortical parvalbumin-expressing (PV+), fast-spiking interneurons have no connection specificity to pyramidal neurons, rather they present as an ’unspecific, densely homogeneous matrix covering all nearby pyramidal cells’ ( 21, p. 13260), which corresponds to our model.

Conditions for neuronal read-out may include the activity of inhibitory neurons. Inhibition and excitation are tightly linked by feedback interaction. Graupner and Reyes (2013) ^22^ suggested that the close coupling of inhibition and excitation in cortical tissue cancels out purely input-dependent, i.e. not network generated synchrony. Rudolph and Destexhe (2003) ^23^ suggested that with highly correlated input, both inhibitory and excitatory, the neuron may receive less input which allows it to be driven only by strong synaptic input, while distributed input consists of a barrage of excitatory and inhibitory inputs where the membrane voltage remains close to firing threshold and the neuron fires continuously. In our sense, it is ’reading out’ its stored intrinsic frequency. Inhibitory and excitatory synaptic input conform to be either asynchronous or synchronous, to drive neurons by correlated input or to cause them to emit spikes according to their own intrinsic frequency.

However, neuromodulation has effects on inhibitory neurons as well ^24, 25^, which we have not modeled. Further simulations will show whether the I-E coupling is altered during enhanced neuromodulation, or whether the effects are synergistic with the present results.

### The role of neuromodulation

Neuromodulation influences both intrinsic properties and synaptic connectivity ^5^, e.g., acetylcholine, (via nucleus basalis stimulation), noradrenaline (via LC stimulation) or dopamine (via VTA stimulation) ^5, 26^. Experimental estimates on the distribution of synaptic neuromodulatory receptors are at approximately 30%–50% of connections ^20^. That is sufficient to transform the topological properties of a graph, such as the width of its degree distribution from heavy-tailed graph to a more Gaussian, less clustered graph without requiring tight optimization for the positions of neuromodulatory receptors ( Figure 12). Neuromodulation disables or enhances various ion channels, such as Sk-channels which guide reset times after a spike, or A-type potassium channels which influence latency to spike ^6, 27, 28^. In this way, neuromodulation influences intrinsic properties ^29^. If neuromodulation reduces synchrony by acting at synaptic receptors, it uncovers intrinsic heterogeneity, and induces a mode of processing that allows read-out and storing of intrinsic properties. Depending on the neuromodulator used, and the amplitude and duration of the signal, different somadendritic ion channel profiles would emerge ^30, 31^.

In the synchronous mode, intrinsic heterogeneities are reduced in the presence of tightly correlated input which drives neurons reliably. This invariance of neuronal intrinsic properties in synchronous mode allows synaptic transmission and information processing independent of neuronal heterogeneity.

The idea of introducing synchronous events by common input to an asynchronous background, and in this way use reliable synaptic transmission without affecting the state of the system (multiplexing) has also been documented in experimental results. For instance, (Gutnisky *et al.*, 2017 ^32^, Figure 4A) shows a case of multiplexing in response to behavioral stimuli. In this case, intrinsic read-out can continue, and single events are transmitted reliably through driven activations.

Why should synchronization properties be switched by neuromodulation? Increased correlation in the network supports population-coded information to be propagated effectively ^33^. Turning on neuromodulation would decorrelate an area and increase the capacity for information coding in an ensemble or a cortical microcolumn ^34^. This area would become an information source to surrounding areas. When turned off, increased correlation would allow this area to transmit information and to disregard the stored neural memory.

### Relation to experimental evidence 

Basal forebrain stimulation, which results in increased acetylcholine release and muscarinic/nicotinic receptor activation, decreases correlation between cortical neurons ( 35– 37 (Figure 3.C)). Likewise, (Minces *et al.*, 2017 ^38^, Figure 3 and Figure 4) shows reduced noise (internal) correlations with cholinergic stimulation, while inactivation of the basal forebrain caused more synchronized activity. Jeanne *et al.*, 2013 ^39^ shows reduction of correlation for task-relevant perception, where presumably task-relevance causes neuromodulatory activity. Fazlali et al., 2016 ^40^ provides evidence for the involvement of noradrenaline in desynchronization of cortical state and the enhancement of sensory coding.

There is considerable evidence ^41– 44^ showing that several neuromodulators, including at least noradrenaline and acetylcholine, modulate pairwise spike correlation, such that strongly synchronized states (anesthesia, slow wave sleep) have high correlation and low neuromodulation, while asynchronous states (normal waking), with higher neuromodulation, have lower pairwise correlation.

Beaman *et al.*, 2014 ^44^ observed intrinsic fluctuations in synchronization of cortical networks during wakefulness which correlated with the amount of encoded perceptual information and perceptual performance. Their results showed a mean decrease in correlations from synchronized to desynchronized state corresponding to perceptual performance by approximately 20%, similar to values observed during attention ^45^, and after adaptation ^46^. We have shown ( Figure 11) that correlation changes are continuous with network topology and a 20% correlation change is well within the range of the current simulations. Importantly, the results in Beaman *et al.*, 2014 ^44^ point to fluctuations in synchronization that reflect local changes in network activity rather than just global cortical state dynamics which have traditionally been associated with central neuromodulatory release.

The role of presynaptic neuromodulation in suppressing cortical connections ^11, 12^ and changing attractor states ^47^, as well as allowing rapid synaptic weight changes ^20^ has previously been assessed. Theoretical work has also emphasized the connection between correlations and information content ^34, 48– 50^.

Here we bring these observations together to suggest that neuromodulation of synapses may alter network topology and in this way bring about an increased decorrelation of spiking, and a more asynchronous state, with a higher informational capacity. It may provide a general explanation (a) on how fluctuations in synchrony can be engineered rapidly and in small cortical areas and (b) why intrinsic memory may be conditional, accessible only at certain times and in a localized fashion.

## Conclusion

We created a number of different parameterized neuron models to capture neuronal heterogeneity. This affects the properties of the neuron such that it has less or more intrinsic excitability, leading to different firing rates when stimulated in an asynchronous way. Under synchronous stimulation the differences are greatly reduced.

We also suggested that synaptic neuromodulation can be an effective way of rapidly altering network topology. We investigated changes in network topology along the dimensions of Gaussian vs. heavy-tailed degree distributions. We hypothesized that heavy-tailed graphs produce more globally synchronized behavior than comparable Gaussian graphs. In accordance with the hypothesis, we find that in a heavy-tailed graph, because of high population synchrony, the difference between neuronal intrinsic properties is minimized, while a Gaussian graph allows read-out of neuronal intrinsic properties. Thus, altering network topology can alter the balance between intrinsically determined vs. synaptically driven network activity.

## Data availability

Underlying data for this study is available from Zenodo. Dataset 1. gscheler/CNeuroSyn: initial version, https://doi.org/10.5281/zenodo.1164096 ^51^.

Data is available under a Creative Commons CC BY-NC 4.0 license.

## Software availability

The source code for the model is available from GitHub: https://github.com/gscheler/CNeuroSyn/ tree/V1.0/src/analysis

Archived source code at time of publication is available from Zenodo https://doi.org/10.5281/zenodo.1164096 ^51^.

Software is available under GNU GPL v2.0 license.
