# Supplementary material for: Neuromodulation influences synchronization and intrinsic read-out
Source: F1000Res. 2018 Dec 2;7:1277. Originally published 2018 Aug 14. [Version 2] doi: 10.12688/f1000research.15804.2 (PMC6426090; doi:10.12688/f1000research.15804.2)
Supplement: Peer review history

# Peer review: Reviewer response for version 2

**DOI:** 10.5256/f1000research.18791.r45036

**Published:** 2019-03-15

**Authors:** Jonathan P. Miller

**Affiliations:** 1 Department of Neurosurgery, University Hospitals Cleveland Medical Center, Case Western Reserve University, Cleveland, OH, USA

The authors of this paper sought to clarify how synaptic efficacy and intrinsic excitability of small-scale neural networks might be affected by neuromodulatory impulses. In a two-dimensional computational model simulating 210 excitatory cortical pyramidal neurons in a cortical microcolumn, they assessed spike synchronization under different conditions of network topology and density in order to assess the relationship of inputs and intrinsic excitability to output. They found that intrinsic network properties influenced output with asynchronous neuromodulatory inputs, whereas synchronous inputs were associated with driving of neural activity such that intrinsic properties disappear. The authors conclude that this effect may underlie flexibility of cortical computations, since the nature of inputs allows networks to shift between transmission and intrinsic readouts based on the synchrony of inputs.

 Neural networks are known to have the ability to perform differently under different input conditions, allowing intrinsic information to be accessed or ignored depending on the state of the network, and this study provides insight into mechanisms underlying these processes. Notably, these observations are somewhat limited by the fact that they are based entirely on simulations with no confirmation in an actual experimental model, so there is a risk that the observed phenomena might be artifacts of assumptions used to build the model in the first place. The simulation is relatively simplistic, consisting of a small-scale model of exclusively excitatory neurons with excitatory connections driven by a single ion channel type. Nevertheless, this information suggests that the frequency specificity of synaptic neuromodulatory might affect synchrony of outputs as assessed by spike rates, and this has intriguing implications for informational capacity and conditional intrinsic memory.

I have read this submission. I believe that I have an appropriate level of expertise to confirm that it is of an acceptable scientific standard.

# Peer review: Reviewer response for version 2

**DOI:** 10.5256/f1000research.18791.r43276

**Published:** 2019-02-19

**Authors:** Krešimir Josić

**Affiliations:** 1 Department of Mathematics, University of Houston, Houston, TX, USA

I will not summarize the work again, as this was already done in the careful reviews of the first two referees. These referees have identified a number of issues that were only partially addressed in the current version of the manuscript. In particular, the generality and interpretation of the results, given that they are based solely on simulations, need to be addressed better, as pointed out earlier. Moreover, a number of relevant references are not discussed, and it is hence not possible to understand how the present manuscript complements the voluminous literature on the subject.

- The \mu in Eq. 1 is a multiplicative factor, from what I gather from ref. 6. This is not clear. 
- Why is \sigma in the exponent in the definition of sigma*?

It is unclear that with N = 210 the effects of different degree distributions can be clearly observed. With this number of neurons finite size effects are likely still strong. In particular, the effects of a heavy-tailed distribution will likely only become apparent with more cells.

- The effects of driving neurons by synchronous input have been noted before, by Selinas and Sejnowski, and many others. As the previous reviewer points out, it is unclear what is different here. Similarly, the hypothesis that hub neurons can synchronize a population has been suggested before, eg. Bonifazi, et al Science, 2009 ^1^, and many others.  
- The currents in Figs 2A and 4B are nearly periodic. I would not expect this, unless they were generated using a mother process. In this case, the observation is actually a consequence of higher order correlations, ie. a common, synchronous input from many neurons that drive a response. Since it was unclear how the correlations in the inputs were generated, I could not tell if this was the case. However, this needs to be clearly explained. 

The first two reviewers have made extensive comments. These were not addressed adequately. I believe these should be resolved before moving forward.

I have read this submission. I believe that I have an appropriate level of expertise to state that I do not consider it to be of an acceptable scientific standard, for reasons outlined above.

## References

[1] BonifaziPGoldinMPicardoMAJorqueraICattaniABianconiGRepresaABen-AriYCossartR: GABAergic hub neurons orchestrate synchrony in developing hippocampal networks. *Science*.2009;326(5958) : 10.1126/science.1175509 1419-24 10.1126/science.1175509 19965761

# Peer review: Reviewer response for version 2

**DOI:** 10.5256/f1000research.18791.r41341

**Published:** 2019-01-08

**Authors:** Joel Zylberberg

**Affiliations:** 1 Department of Physiology and Biophysics, University of Colorado School of Medicine, Aurora, CO, USA

I appreciate the revisions, and I think that the new version is clearer and more complete than the previous one.

  I still have a few minor concerns/suggestions: addressing these will, in my opinion, enhance the clarity and impact of this work.

- I still think it’s important to mention in this paper (ideally in the Methods section) that the modelled synaptic inputs come from a current-based synapse model. That information is present in Ref. 6 of course, but it should be here for completeness. That way, without reading Ref. 6, readers will be able to understand why all the neurons (e.g., in Fig.1) get the same input current trace, even though they have different voltage time series: with conductance-based synapses, identical synaptic conductance inputs would lead to different current input time series.
- Some of the claims should be softened a bit, in my opinion. In so doing, they would be better supported by the data that’s shown. 

For example, in reference to Fig. 2 (p. 5): “We stimulate the same neurons with the correlated input and observe the spike pattern (Figure 2). We can show that the frequency-specificity of the neuron disappears.”. This isn’t quite what the data show: the data show a strongly reduced difference in ISI distributions (vs. Fig. 1), but there is still a remaining difference. So it’s not the case that the difference has “disappeared”.

 The same comment applies to the discussion of Fig. 5 (top of p.6): “the ISIs become identical, but with a wider distribution to reflect the different duration of pauses between the synchronous stimulation”. The ISI distributions shown in Fig. 5 are very similar, but they aren’t quite *identical*. So I’d suggest softening the language a bit in these places, to be more precise about what’s actually shown. I don’t think these changes much affect the conclusions of the paper, but they do make the text more accurate.

 The discussion of Fig. 4 in the text is a good example of what I’m recommending: there, the distributions are described as *nearly* identical, which is a fair description at least for cells 1,2,4,5.

- Bottom of p. 8, “We now stimulate the graph by an initial stimulation to 10 randomly selected      excitatory neurons (for about 1 second).” I still think it’s important to be clear about the protocol used for this stimulation. Was it with constant current injection? Was it with fluctuating current traces mimicking asynchronous inputs like in Fig. 1A, or with fluctuating current input mimicking synchronous input like in Fig. 2A? Moreover, was it the same current input for all neurons, or did they have different current inputs? Providing this information will give the reader context to understand and evaluate your methods, and the corresponding findings.

I have read this submission. I believe that I have an appropriate level of expertise to confirm that it is of an acceptable scientific standard.

# Peer review: Reviewer response for version 1

**DOI:** 10.5256/f1000research.17251.r38207

**Published:** 2018-10-24

**Authors:** Guillaume Drion

**Affiliations:** 1 Department of Electrical Engineering and Computer Science, University of Liege, Liège, Belgium

This paper compares (i) the response of heterogeneous neurons to two different types of synaptic inputs (uncorrelated or correlated), and (ii) the response of neuronal populations having two different types of synaptic weight distribution (Gaussian or Lognormal). The main results can be summarized as follows:

1. The chosen neuronal heterogeneity induces variability in mean spiking frequency. This variability persists under the application of uncorrelated synaptic inputs, but is masked by the application of correlated synaptic inputs.
2. Stimulating a subpopulation of neurons in a neuronal population induces an asynchronous response when the distribution of synaptic weights is Gaussian, whereas it induces a synchronous response when this distribution is Lognormal.

Then, it is speculated that switches between asynchronous and synchronous states can be controlled by neuromodulators, which would modify the network topology from Gaussian to Lognormal and conversely.

 After carefully reading this paper, I think that neither the content nor the form is in a sufficiently advanced stage for the manuscript to be indexed in its current form. A thorough revision is strongly suggested to boost the potential impact of the research. I provide specific comments below, in order of appearance in the text.

Table and figure captions are not descriptive enough. One cannot fully comprehend the different figures, which include numerous notations, by reading the captions. Some notations are not consistent (in Fig. 1-6, ISI is noted I=*** on the left panels, and ISI on the right panels).

Title: The title is totally misleading. It states that “neuromodulation influences and intrinsic read-out”, but nothing of the sort is demonstrated in the manuscript. Neuromodulation is only speculated to be a plausible source to control the mechanisms highlighted in this manuscript, without any evidence or results to support this speculation. I have nothing against speculations and interpretations, quite the contrary, but they should not appear as grand truth in the title.

Abstract: The “Methods” part of the abstract is solely composed of results. This part should summarize the methods used in this paper.

Methods:

 “ *For the experiments in this paper, we only focus on variability induced by changes in the strength of the slow A-type K channels. The total current contribution for this channel is μIAs where μ was selected between 1.0 and 1.5." *Please motivate this choice (both channel type and chosen range), because the model is composed of 6 voltage-gated channels that could be used to model variability. Also, I would not use “experiments” but “simulations”, although this is a minor comment.

 “ *Time-dependence is modeled by the gating variable u.*” What does this sentence mean? Both ODEs model time-dependent evolution.

 “ *By varying d, we can vary the inactivation dynamics of the model after a spike, by varying a we vary the inactivation dynamics throughout the computation.” *I do not see the why variables *a* and *d* correspond to inactivations. *a* is the time constant of activation/deactivation of *u* ( *u* indeed activates/deactivates, the slope *b* being always positive), and *d* is the increase in *u* after a reset, which represents how much a spike activates *u*.

 “ For the excitatory neurons, we use randomly connected graphs (N,K) with different dispersion sigma*. This corresponds to normal (Gaussian) to lognormal graphs with different widths and length of the heavy tail.”. This dispersion coefficient, and how it “corresponds to normal (Gaussian) to lognormal graphs with different widths and length of the heavy tail “, should be defined properly, because it is an important parameter in the results.

 “ *The rewiring algorithm used to change the properties of a graph G is a greedy algorithm, which iteratively selects the node s with the highest degree*”. Does “ *s*” refer to a node, or is it a measure of network synchronization?

Results:

*“We show how we can model gain as a stored intrinsic property, defined as a spike rate in response to constant or fluctuating input of fixed strength (A/Hz)” *This sentence is unclear. What kind of gain is it referring to? What is a “stored” intrinsic property? What does “fixed strength” mean for a fluctuating input?

 “ *In Figure 1A, we show the response of MSN model neurons with a scaling of μIAs = 1.0, 1.3, 1.5 to a noisy signal, derived from uncorrelated Poisson-distributed synaptic input.”* From what I understood, neurons are not connected to each other in this case. It should be explicitly mentioned, because at this stage it is unclear whether we discuss isolated neurons or neurons within a network.

 “ *The total number of spikes includes bursts, which were excluded from ISI calculation.*” How were bursts defined? Why were they excluded from ISI calculation? But then why excluding them from one measure but not from the other? 

*Results related to Fig. 1 and 2*: Only one example of uncorrelated/correlated input is shown, and conclusion is drawn from this example. This part requires further investigation. For instance, the increase in firing rate that we see in Fig. 2 could potentially induce overlapping of spiking rate, because it moves the neurons to a flatter portion of the f-I curve. It is important to rule out this effect to make sure that the difference is indeed due to the correlated/uncorrelated nature of the inputs, for instance by showing that increasing the firing rate with uncorrelated synaptic inputs does not induce the same effect. I also do not find surprising that inputs with high amplitude, low frequency fluctuations would affect the firing more than an almost constant input, but I guess that it is more subjective. You also talk about “a time-locked spike pattern”. How do you define it in the context of this paper? If is it synonymous of synchronization, having clearly separated frequencies does not mean neurons cannot be synchronized: one neuron could have a frequency that is twice the other and be fully synchronized, yet the frequencies would be separated. On the other hand, two neurons could fire at a same frequency and coefficient of variation, and yet not be synchronized.

*“To continue with exploring this property of model neurons in a networked context, we switched to a simplified model neuron7 and created a set of variations for this model (see Methods).”* Again, I guess that the results of this section are provided on isolated neurons. If it is the case, this sentence is misleading.

*Results connected to Fig. 3, 4 and 5*: My comment connects to the one relates to Fig. 1 and 2. The spikes of Fig. 3A look much more “time-locked” than the ones of Fig. 2A, although the conclusions that are drawn from these two figures are opposite. Is the “clear separation of ISIs” such a good measure in this context? In Fig. 4 and Fig. 5, most events look like bursts. Are bursts excluded from ISI calculation like in the previous case? If not why? Again, how is bursting defined in this case as compared to the previous case? Finally, in Fig. 5 all neurons seem to burst, yet the distribution of ISIs is a Gaussian, which does not make sense, unless you are only plotting the distribution for the interburst intervals.

 “ *We first use a Gaussian connected graph (RG) with N = 210 and K = 1800 and use 7 different neuronal types (1–6, plus the generic neuron g) with 30 Neurons each (Methods). Figure 7A shows an excerpt of the graph structure. We can see that the graph is connected such that all neurons have a comparable number of connections. This is also apparent in Figure 8, where we can see a (narrow) normal distribution for connectivity for the Gaussian graph RG. Table 2 contains the graph characteristics for both graphs.” *Only one graph is mentioned in the paragraph, and Table 2 sows 5 different cases. Both graph probably mean “Gaussian” and “Lognormal”, but that should be clarified.

 “ *We now stimulate the graph by an initial stimulation to 10 excitatory neurons (for about 1s).*” How were these 10 neurons selected? Where they selected randomly, or based on their degree of connectivity? Are they chosen to correspond to nodes with a high connectivity degree in the lognormal case? If so, then what is happening if we either stimulate neurons with lower connectivity degrees in the lognormal case, or add a few hub neurons in the Gaussian case? Is the difference in synchronization really due to the whole distribution of the synaptic weights, or simply due to the existence of hub neurons that would be specifically targeted by synaptic inputs? This question needs to be answered in order to support the conclusion “ *In our model, the pairwise synchronicity s is dominated by the network topology, more precisely by the width of the degree distribution (dispersion) ranging from Gaussian to lognormal with a heavy tail.”*

 In Figure 11, are the “experimental conditions” similar than in Figures 9 and 10? Which 10 neurons are stimulated?

Discussion

 “ *Neuromodulation disables or enhances various ion channels, such as SK-channels which guide reset times after a spike, or A-type potassium channels which influence latency to spike.” *References that support these claims are needed.

I have read this submission. I believe that I have an appropriate level of expertise to confirm that it is of an acceptable scientific standard, however I have significant reservations, as outlined above.

# Peer review: Reviewer response for version 1

**DOI:** 10.5256/f1000research.17251.r37164

**Published:** 2018-08-28

**Authors:** Joel Zylberberg

**Affiliations:** 1 Department of Physiology and Biophysics, University of Colorado School of Medicine, Aurora, CO, USA

This study asks when the intrinsic biophysical properties of neurons do or do not affect their spiking responses to synaptic inputs. I denote the main claims and results of the paper as I,II,and III below, each of which is discussed individually thereafter.

 I. The author begins by noting that, in the presence of strong synchronous inputs, essentially all neurons are driven to quickly spike, regardless of their intrinsic properties. In the presence of less-synchronous inputs, differences in intrinsic properties do lead to noticeable changes in spiking outputs. This is (presumably) because differences in integration time, and excitability (effective spiking threshold) determine how much input is needed to get the cell to spike, but sufficiently strong synchronous inputs always suffice to generate spikes. This is a neat observation, somewhat obvious in hindsight, but nevertheless made me glad to read the paper.

 II. Next, they investigate how different network structures (quantified by dispersion of the graph's out-degree distribution) affect the synchrony in the inputs to individual neurons: networks with high dispersion (e.g., small numbers of critical "hubs" in the network) lead to higher synchrony. Thus, in those networks, the neurons' intrinsic properties are less influential than in networks with lower dispersion.

 III. Finally, the authors speculate that, because neuromodulators can change effective connectivity, they could switch the network between different graph structures, thereby either enabling, or disabling, the influences of neurons' intrinsic properties. In that way, the network can "multiplex" between operational modes, on of which (the asynchronous one) is more affected by neurons' intrinsic plasticity, and thus more affected by memory and experience.

 Below, I summarize first the relation to prior studies for each claim (I-III), and then comment on some technical and presentation issues that, to me, hinder somewhat the readability of the paper. My hope is that addressing these concerns will help the author to increase the impact of their work.

Comments on relation to other studies:

Result I. This is a neat observation. It is somewhat foreshadowed by some older work by Salinas and Sejnowski ^1^^,^^2^, showing that synchronous inputs are good at driving spikes in basically any neuron model. It would be useful to differentiate the findings of this paper from their older work.

Result II. Closely related results were obtained in a pair of studies by Yu Hu, James Trousdale, and colleagues ^3^^,^^4^. There, they computed the correlation structure (closely related to the author's synchrony measure) in networks with different connectivity motifs, using a neat analytical approach involving motif cumulants. It would be worthwhile (again) differentiating the results here from that prior work, so that readers know what's new and significant about this paper vs. the prior state of the field.

Result III. I'm puzzled by the claim that the network topology is strongly affected by neuromodulators (which is the key to the switching property that's discussed through the paper). While I understand that neuromodulators can strengthen or weaken synapses, that mechanism will change the weights within the graph describing network connectivity, but not change the underlying unweighted graph describing which neurons are connected to each other (regardless of the strength of that connection). To change the network topology, the neuromodulators would need to either a) add new synapses (or "unsilence" previously silenced synapses) selectively to some neurons, or b) remove (or completely silence) synapses selectively from some neurons. I think that, in order for the author to make claims about neuromodulators changing network topology, they should include some discussion (ideally based on the experimental literature) of when and how neuromodulators actually change network topology. This literature could of course exist: there's lots that I don't know about neuromodulators. And in that case, I think the author's claims would be much strengthened by referencing and discussing it.

Comments on presentation and specific technical issues:

1. Methods, 3rd paragraph. Why only focus on modulators affecting slow A-type K channels? Are those the most important ones? The ones most affected by common neuromodulators? Which neuromodulators are those? 
2. Methods, 4th paragraph. It would help me if you would describe explicitly the method used to generate either correlated or uncorrelated synaptic input traces. For example, do these incoming spike trains (generating the synaptic inputs) come from a process like SIP or MIP? And specifically, how were the "correlation factors" quantified and manipulated?
3. Similar to (2) it would help to describe explicitly the synapse model used. For example, are the synapses conductance based (e.g., convolve incoming spike trains with a double exponential to get conductance traces)? I assume not, given that in Figs. 1-6, the same synaptic current trace is shown for all neurons. If the synapses were conductance-based, the differences in membrane potentials of those neurons would lead to differences in synaptic currents. 
4. The definition of the synchronization is fine to me but doesn't seem to match the description in the preceding paragraph. Specifically, the formula doesn't seem to "count the number of other neurons which fire in a 10ms window. . . " so much as "count the number of spikes emitted by each other neuron in a 10 ms window".
5. I'd describe the rewiring process in the "graph properties" section of the Methods and not the "Definitions" section. That way, when you introduce the dispersion \sigma, you can explain the process used to manipulate it in your simulations. 
6. The figures generally could use much more annotation, to help the readers parse what all the different curves mean. For example, in Figs. 1 and 2, I'd color code the curves (distributions) in panel B, and have those same colors used for the different spike trains in panel A, so that readers know which distribution goes with which spike train goes with which model. The same idea could be applied to the other figures: help the reader out so that, at a quick glance (which is how people read papers now) they can tell what's what and what the main point is.
  Similarly, in Fig. 11, I'd label the color bar, so that people know what variable the colors are describing. 
7. The formula for density on p. 6 (bottom left) seems incorrect, as d=K/(NxN-1). That would be K/(N^2 -1), whereas I think you mean K/[Nx(N-1)]. 
8. P. 6, upper right, description of stimulating the graph by driving 10 excitatory neurons. How was this done? Did you inject current into those model neurons? If so, how much?
9. Some consistency between the network sizes in the simulations would help a lot. Specificlaly, in Fig. 10, you study a 1924 neuron network, whereas Fig. 9 uses 1800 neurons. This leave the interpretation muddier than it needs to be: readers might wonder whether some of the differences could be attributed to changes in network size, and not just to changes in connectivity. In general, I'd suggest keeping all but one key variable constant, so that the comparisons can be made as cleanly as possible.

I have read this submission. I believe that I have an appropriate level of expertise to confirm that it is of an acceptable scientific standard, however I have significant reservations, as outlined above.

## References

[1] SalinasESejnowskiT: Impact of Correlated Synaptic Input on Output Firing Rate and Variability in Simple Neuronal Models. *The Journal of Neuroscience*.2000;20(16) : 10.1523/JNEUROSCI.20-16-06193.2000 6193-6209 10.1523/JNEUROSCI.20-16-06193.2000 PMC677257410934269

[2] SalinasESejnowskiTJ: Correlated neuronal activity and the flow of neural information. *Nat Rev Neurosci*.2001;2(8) : 10.1038/35086012 539-50 10.1038/35086012 11483997PMC2868968

[3] TrousdaleJHuYShea-BrownEJosićK: Impact of network structure and cellular response on spike time correlations. *PLoS Comput Biol*.2012;8(3) : 10.1371/journal.pcbi.1002408 e1002408 10.1371/journal.pcbi.1002408 22457608PMC3310711

[4] HuYTrousdaleJJosićKShea-BrownE: Local paths to global coherence: cutting networks down to size. *Phys Rev E Stat Nonlin Soft Matter Phys*.2014;89(3) : 10.1103/PhysRevE.89.032802 032802 10.1103/PhysRevE.89.032802 24730894
